# How Well Do Computer-Generated Faces Tap Face Expertise?

**DOI:** 10.1371/journal.pone.0141353

**Published:** 2015-11-04

**Authors:** Kate Crookes, Louise Ewing, Ju-dith Gildenhuys, Nadine Kloth, William G. Hayward, Matt Oxner, Stephen Pond, Gillian Rhodes

**Affiliations:** 1 ARC Centre of Excellence in Cognition and its Disorders, School of Psychology, University of Western Australia, Perth, Australia; 2 Department of Psychological Sciences, Birkbeck, University of London, London, United Kingdom; 3 Department of Psychology, University of Hong Kong, Hong Kong, China; 4 School of Psychology, University of Auckland, Auckland, New Zealand; Vanderbilt University, UNITED STATES

## Abstract

The use of computer-generated (CG) stimuli in face processing research is proliferating due to the ease with which faces can be generated, standardised and manipulated. However there has been surprisingly little research into whether CG faces are processed in the same way as photographs of real faces. The present study assessed how well CG faces tap face identity expertise by investigating whether two indicators of face expertise are reduced for CG faces when compared to face photographs. These indicators were accuracy for identification of own-race faces and the other-race effect (ORE)–the well-established finding that own-race faces are recognised more accurately than other-race faces. In Experiment 1 Caucasian and Asian participants completed a recognition memory task for own- and other-race real and CG faces. Overall accuracy for own-race faces was dramatically reduced for CG compared to real faces and the ORE was significantly and substantially attenuated for CG faces. Experiment 2 investigated perceptual discrimination for own- and other-race real and CG faces with Caucasian and Asian participants. Here again, accuracy for own-race faces was significantly reduced for CG compared to real faces. However the ORE was not affected by format. Together these results signal that CG faces of the type tested here do not fully tap face expertise. Technological advancement may, in the future, produce CG faces that are equivalent to real photographs. Until then caution is advised when interpreting results obtained using CG faces.

## Introduction

Advances in technology have seen an increase in the use of computer-generated (CG) stimuli in face processing research in recent years. Artificial faces with a very human-like appearance can now be generated by a number of software programs with ease (either ‘from scratch’ or by inputting real photographs to be converted into 3-D head models). Different facial characteristics can be specified or varied when generating these faces including sex, age, ethnicity and attractiveness. Once generated, the faces can then be easily manipulated for facial expression and viewpoint. CG faces are also highly standardised in terms of lighting conditions, extra-facial information, size and image quality. All these factors make CG faces very appealing to face processing researchers, particularly given the limitations that existing databases of face photographs often impose on experimental design and the cost and time required to generate new photographic databases. However little is known about the validity of the CG faces being used in research, and it remains unclear whether, as stimuli, they are equivalent to photographs of real faces.

Humans are generally considered face experts, demonstrating remarkable abilities to extract a range of social information from faces. Despite little evidence regarding their validity, CG faces are being used to address important questions in face processing research. Examples include charting the developmental trajectory of face identity recognition [1], exploring the origins of race effects on face recognition [2], identifying the perceptual underpinnings of social judgements from faces such as trustworthiness [3], mapping the structure of face-space [4], investigating the types of faces for which there is special sensitivity to spacing between features in upright faces [5], and examining the category selectivity of neural responses to faces [6]. Results from such studies are being used to inform our understanding of how faces are processed and to develop and refine theories of face processing. It is therefore critically important to know the extent to which the CG faces being used in these studies truly allow for the demonstration of face expertise.

As the above examples attest, CG faces are being used to study a broad range of face processing abilities. The present study focussed on one aspect of face processing: namely the expert processing of face identity. Given the similarity between faces, our ability to efficiently discriminate between identities and accurately recognise many hundreds of familiar individuals is truly remarkable. It is generally agreed that this ability is supported by specialised face processing mechanisms (for review see [7]). However, this expertise is sensitive to deviations from the types of faces we are used to dealing with. For example people tend to demonstrate greater expertise, in the form of greater recognition accuracy, for faces of their own race than for faces from other races (for review see [8]). CG faces may represent another category of faces with which we are less expert.

**Fig 1:**
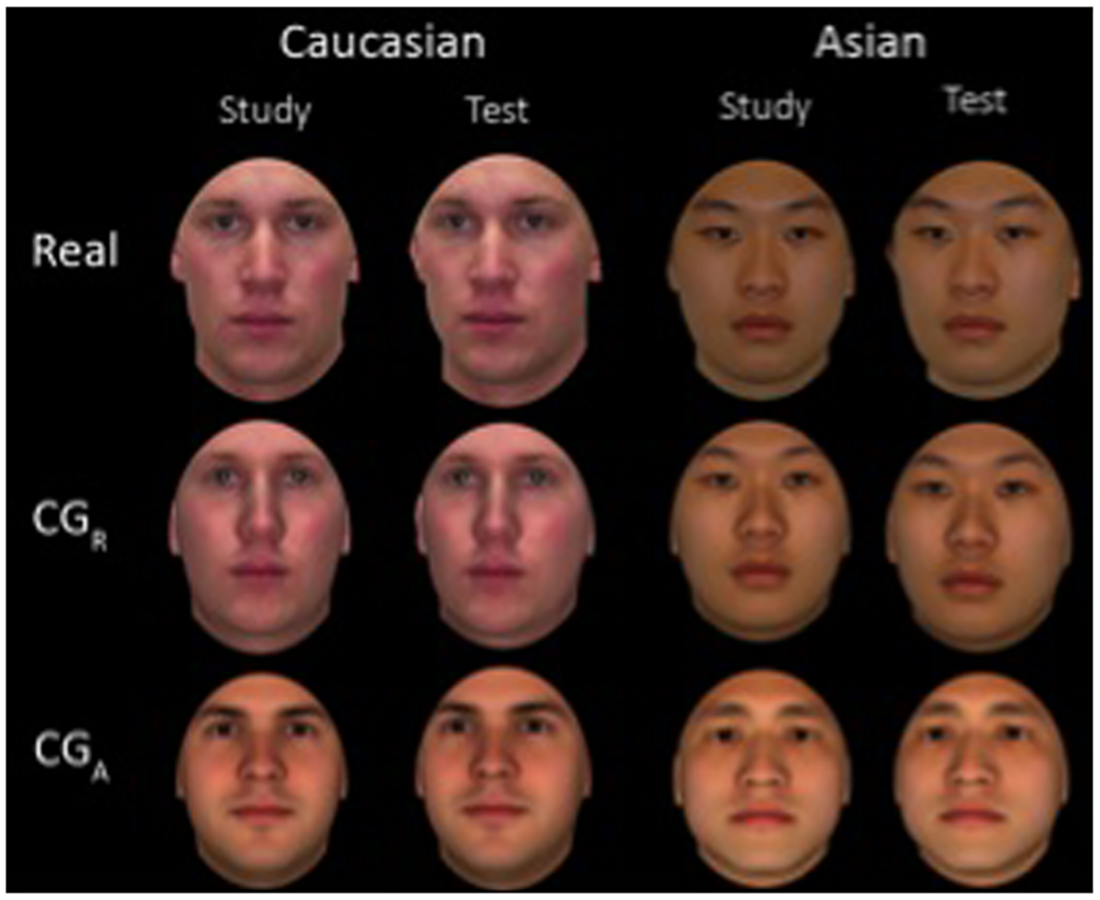
Example Caucasian and Asian stimuli in the three formats used in Experiment 1: Real, CG_R_, CG_A_. A slight view change was included between study and test (i.e., study faces = front view, test faces = 5° left or right). Note the same identities are depicted in the Real and CG_R_ conditions.

CG faces that are currently being used in research are commonly generated by a program called FaceGen. They are remarkably human-like (see Fig 1 for examples), but they are certainly distinguishable from, and less familiar than, real photographs, which could potentially reduce their ability to engage face expertise. The most notable difference between these CG faces and face photographs is that the CG faces appear to lack fine-grained surface texture information and imperfections that are usually present in photographic face stimuli. This gives the impression of these faces being somewhat artificial and unreal. These CG faces may also lack animacy—the perception that a face belongs to a living being with a mind [9, 10]. Recent studies have found that behavioural and neural responses to faces are highly sensitive to animacy [10–13]. These clear differences raise the question of whether CG faces are processed in the same way as real faces and can allow for the full demonstration of face expertise.

Beyond giving the CG faces an unnatural appearance, the visible loss of surface texture information in these CG faces may also indicate a more primary issue with such stimuli. It could be that these faces lack vital information that is used by our face processing systems to recognise and discriminate faces. There is evidence that surface texture information is important for face recognition (e.g., [14, 15]). Similarly structural shape information (e.g., [14]) and information in certain frequency bands (e.g., [16–18]) have also been shown to be vital for recognition. If such diagnostic information is impoverished or absent in CG stimuli then they may not fully tap expert face recognition mechanisms.

In the area of identity recognition three studies have addressed the question of whether CG faces are processed like real faces. All three used FaceGen to generate stimuli. Matheson and McMullen [19] found that three hallmarks of face identity expertise—the other-race effect (ORE), the inversion effect and the reduction in the inversion effect for other-race compared to own-race CG faces—were present for randomly generated CG faces. These three effects reflect the fact that people tend to have the greatest expertise for, and therefore tend to be most accurate at recognising, upright own-race faces. They are less accurate at recognising faces presented upside-down (e.g., [20]) or faces from another race [8]. Given that people are less expert with other-race faces than own-race faces, inversion also has less of an effect on other-race faces [21–24]. Having demonstrated these key effects, Matheson and McMullen [19] concluded that CG faces are processed in a similar way to photographs of real faces and are therefore suitable for use in face research. Critically, however, methodological issues with their study permit other possible interpretations of the observed patterns.

First, the major critique, which affects the interpretation of all three results, is that a real face (i.e., photograph) condition was not included in the experiment. Therefore, it is impossible to know whether the observed effects were of the same strength as those that would be observed for real faces. The results do suggest qualitatively similar processing of real and CG faces. However to conclude that CG faces are equivalent to real photographs and allow for the full demonstration of face expertise it is necessary to not only qualitatively demonstrate key ‘expertise effects’ but also to show that these effects are quantitatively as strong for CG faces as for real faces. Second, only Caucasian participants were tested. This leaves open the possibility that rather than being an expertise effect, the observed ORE might have reflected stimulus effects, that is, the African American faces created for the study might have simply been more difficult to recognise than the Caucasian faces used. To rule out this possibility it is necessary to demonstrate the (reversed) ORE for the same stimuli with a group of African American participants. The results of Matheson and McMullen’s [19] study therefore do not provide clear evidence of full expert processing of CG faces. Additionally, Matheson and McMullen [19] did note that overall performance was particularly poor on their task (i.e., d' < 1.5). Their interpretation of this result was that the lack of distinctive elements (e.g., skin imperfections) in the CG faces poses a challenge to the visual system. This poor performance may in fact indicate a failure of CG faces to fully engage face expertise.

Papesh and Goldinger [4] also addressed the question of whether an ORE is observed in recognition memory for CG faces in the course of validating stimuli for another study. Here, performance for CG and real faces was directly compared, but the CG faces had been created from the real photographs rather than randomly generated. There was no indication that the ORE was any smaller for CG than real faces. Papesh and Goldinger [4] took this as evidence that CG faces were appropriate substitutes for real face photographs. However, as in Matheson and McMullen [19], only one race of participants was tested, leaving open the possibility that difference between own- and other-race faces was a stimulus effect rather than an expertise effect. Memory accuracy was also numerically poorer for CG than real faces, which might again potentially reflect a lack of expertise for CG faces. Therefore the critical question of whether CG faces fully quantitatively recruit expert face processing mechanisms remains open.

More recently Balas and Pacella [25] compared performance for photographs of real faces to CG versions of the same faces on a recognition memory and a face matching task. They reported that recognition memory accuracy was significantly worse for CG faces compared to real faces and concluded that CG faces are harder to remember. On the face matching task performance was also significantly poorer for CG compared to real faces but this effect was very small (<2%). Importantly, counter to the notion of diminished expert processing for CG faces, Balas and Pacella [25] found no reduction in the size of the inversion effect for the discrimination (inversion was not tested for the memory task) of these stimuli compared to real faces. However accuracy on this task was exceptionally good even in the inverted conditions (approximately 90%), which may have precluded identification of a larger inversion effect in the real faces condition.

There are a number of potential indicators of face identity expertise. The present study investigated two important markers that have been identified in the previous literature: accuracy of own-race face recognition and the ORE. These markers of expertise were tested with regards to both recognition memory (Experiment 1) and perceptual discrimination (Experiment 2). We compared performance with CG faces to that with photographs of real faces in order to detect any potential reduction in own-race accuracy or in the ORE. We also tested both Asian and Caucasian participants with Asian and Caucasian face stimuli to rule out differences between face sets as the source of any ORE. Importantly, unlike the previous three studies [4, 19, 25] which all used identical images at study and test, here we included a viewpoint change to ensure we were testing higher-level face recognition rather than low-level image matching.

If CG faces are equivalent to real photographs and allow participants to fully demonstrate their face expertise, then we expect no differences between CG and real faces in either own-race face accuracy or the magnitude of the ORE. However, if CG faces fail to fully recruit face expertise, then we expect to observe a reduction in own-race face accuracy and reduction of the ORE for CG faces compared to real photographs. This prediction for the ORE is based on the idea that any reduction in expertise would have the greatest effect on the faces we are most expert with—own-race faces. It may even be the case that CG faces do not recruit face expertise at all, in which case we would expect the ORE to be eliminated.

## Experiment 1: Recognition memory

In Experiment 1 we tested old/new recognition memory for Caucasian and Asian faces presented in three formats: Real face photographs, CG-Real (CG_R_) faces and CG-Artificial (CG_A_) faces. The two CG formats were chosen because they represent the two types of CG faces that have been used in previous studies. CG_A_ faces were randomly generated by the software. This type of CG face is the most common in the literature (e.g., [2, 19, 26–28]). CG_R_ faces were generated by importing the photographs from the Real condition into the software to produce CG versions of the Real faces, (e.g., [4, 25]). Including both CG formats provides a thorough test of the usefulness of CG face stimuli. CG_R_ faces may also provide a fairer comparison to the Real faces than the arbitrarily generated CG_A_ faces. Assuming 100% fidelity in the conversion process the CG_R_ faces should be matched to the Real faces for within-set heterogeneity. As can be seen in Fig 1 the CG_R_ faces retain some of the imperfections of the Real faces, but still lack some fine-grained texture information and may give a weaker impression of animacy. Texture information was not applied to the CG_A_ faces as this has not been routinely done in previous studies. The CG_A_ faces therefore have a uniformly smooth appearance.

To recap, if CG faces do not fully recruit face expertise then we expect recognition of own-race faces to be less accurate for CG than Real faces and the ORE in recognition memory to be reduced for CG faces.

## Method

### Ethics Statement

The study was approved by the Human Research Ethics Committee at the University of Western Australia and the University of Hong Kong. All participants provided written consent prior to their participation in the project.

### Participants

Caucasian participants were 36 students (17 male; Age: Mean = 20.5, SD = 3.9) at the University of Western Australia. Asian participants were 35 students or staff (10 male; Age: Mean = 20.5, SD = 1.9) at the University of Hong Kong. Participants received either course credit (Caucasian participants) or HK$40 (approximately US$5) for the 40 minute experiment.

### Stimuli

There were three different formats of face stimuli: Real, CG_R_, CG_A_. Each format consisted of 80 young adult males (40 Caucasian and 40 Asian) with neutral expressions (see Fig 1 for examples). There were two versions of each face, one in front view and one facing 5° left or right.

#### Real faces

The 40 Caucasian faces were photographs taken at the University of Western Australia. The 40 Asian faces were all ethnically Chinese and photographed in Hong Kong [29].

#### CG_R_ faces

CG versions of each of the faces from the Real condition were created using the “Photofit” function of FaceGen Modeller 3.5.3. This process involved digitally placing markers at landmark points (e.g., bridge of nose, corner of mouth) on the front and profile views of the original faces (11 markers for front view, 9 markers for profiles). These points were then used to import the face into FaceGen integrating information from the front and profile views to create a 3D model of the head from which 2D images were exported.

#### CG_A_ faces

FaceGen was also used to randomly generate a set of 40 Caucasian and a set of 40 East Asian young adult (approx. 25), male faces. The gender and age settings were locked at the same levels for all faces across both races of face. All faces had the same lighting conditions and no texture information was applied. Controllers for expressions, muscle modifiers (e.g., brow position) and phonemes (i.e., mouth shape) were set to zero (i.e., neutral expression).

The stimuli were edited and standardised using Adobe Photoshop CS3. All the faces were resized to have an inter-pupil distance of 80 pixels. Hair (and, in the CG conditions, bald head) information was masked with a black oval. Chin shape and some neck information was retained but all clothing was masked. None of the faces had facial hair and the stimuli were edited to remove any obvious distinguishing marks (i.e., blemishes, scars, moles). All faces were presented in colour. At the viewing distance of approximately 50 cm stimuli subtended a visual angle of approximately 5.4° × 6.6°.

Real and CG_R_ formats consisted of the same face identities, which were split into two sets of 20 faces for each race (i.e., Set A and Set B) such that Set A contained the same identities in each format. Each participant saw one set (e.g., Set A) in the Real condition and the other set (e.g., Set B) in the CG_R_ condition. Assignment of the face sets to conditions was counterbalanced across participants. For consistency, the faces in the CG_A_ condition were also split into two sets (e.g., Set C and Set D). Half the participants saw one set (e.g., Set C) and the other half saw the other set (e.g., Set D).

To ensure that the task tapped face recognition rather than picture recognition there was a slight viewpoint change between study and test. At study all faces were presented in front view. At test the faces were shown facing 5° to the left or right. Of the old faces, half faced to the right and half to the left. Similarly half of the new faces faced to the right and half to the left.

### Procedure

Stimuli were presented using SuperLab 4.0 (Cedrus Corporation, California) on 21.5 inch iMac computers. Both face format (Real, CG_R_, CG_A_) and race of face (Caucasian, Asian) were blocked and manipulated within participants. Each participant, therefore, completed 6 study-test cycles. The three format blocks for each race of face were completed consecutively (e.g., Asian Real, Asian CG_R_, Asian CG_A_,Caucasian Real, Caucasian CG_R_, Caucasian CG_A_) with the race of face that was completed first counterbalanced across participants. Order of format blocks was counterbalanced across participants according to a Latin square.

Participants were informed that the task would test their memory for faces. They were instructed to concentrate on the study faces carefully because they would see different versions of the faces at test. Within blocks, each study phase was initiated by the participant via a key press. In each study phase, 10 front-view faces were presented sequentially in the centre of the screen for 3000ms each. Each face was followed by a blank screen for 500ms. The order in which the faces were presented was randomised for each participant. The same study faces were then presented a second time (3000ms each), in a different random order. Immediately following the study phase, participants initiated the test phase with a key press. In the test phase, 20 faces (10 “old” studied faces, 10 “new” unstudied faces) were presented sequentially and remained on-screen until response. Participants pressed labelled keyboard keys to indicate whether they thought each face was “old” or “new”. Responses immediately triggered the next trial. Test faces appeared in a different random order for each participant. To familiarise participants with the procedure they first completed a practice block consisting of 6 study and 12 test faces. Practice faces were characters from television cartoon *The Simpsons*.

Following the memory experiment participants completed a racial background and contact questionnaire adapted from Hancock and Rhodes [21]. Participants reported their ethnicity and rated their agreement with 7 statements about each race (e.g., “I know lots of Asian [Caucasian] people”) on a 6-point scale (1 = very strongly disagree; 6 = very strongly agree). Finally participants’ experience with CG faces was assessed using a questionnaire developed for this study. Participants rated their level of agreement with 5 statements (e.g., “I play video and/or computer games that contain computer generated faces) using the same 6-point scale as in the race questionnaire.

## Results and Discussion

### Contact

Self-reported contact with own-race, other-race and CG faces was calculated as the mean of the contact ratings for each type of face (see Table 1). As expected both groups of participants reported significantly greater contact with own-race than other-race faces: Caucasian participants, *t*(35) = 9.85, *p* <.001,Cohen’s*d*= 1.64; Asian participants, *t*(34) = 7.23, *p* <.001,Cohen’s *d* = 1.22. There was no difference between the Caucasian and Asian participants in reported experience with CG faces, *t*(64.9) = 0.43 *p* = .672,Cohen’s *d* = 0.10.

**Table 1:** Experiment 1: Mean (SD) self-reported contact with own-race, other-race and CG faces.

	Own-race	Other-race	CG
Caucasian participants	5.4 (0.6)	3.4 (0.9)	2.8 (1.5)
Asian participants	5.1 (1.2)	2.5 (1.1)	2.9 (1.1)

### Recognition accuracy

Accuracy was measured for each condition using the signal detection measure d' (see Table 2), calculated according to the standard formula d' = z(hits)–z(false alarms). We defined hits as correctly responding “old” to studied items and false alarms as incorrectly responding “old” to unstudied items. Hit and false alarm rates of 0 and 1 were replaced using the conventional formulas 1/(2N) and 1–1/(2N) respectively, where N is the maximum number of hits or false alarms [30]. Proportions of hits and false alarms are available in the supplementary materials (Table A in S1 File). The following analyses address the questions of whether own-race face recognition and the ORE are reduced for CG faces in turn.

**Table 2:** Mean (SD) face recognition accuracy (d') as a function of participant race, race of face and face format.

Format	Real	CG_R_	CG_A_
Caucasian Participants			
own-race faces	1.7 (0.9)	1.4 (0.8)	0.7 (0.8)
other-race faces	1.3 (0.8)	0.9 (0.8)	0.7 (0.7)
Asian Participants			
own-race faces	1.8 (0.8)	1.2 (0.7)	0.8 (0.8)
other-race faces	0.8 (0.7)	1.0 (0.7)	0.4 (0.7)

### Are own-race CG faces recognised less accurately than real photographs?

**Fig 2:**
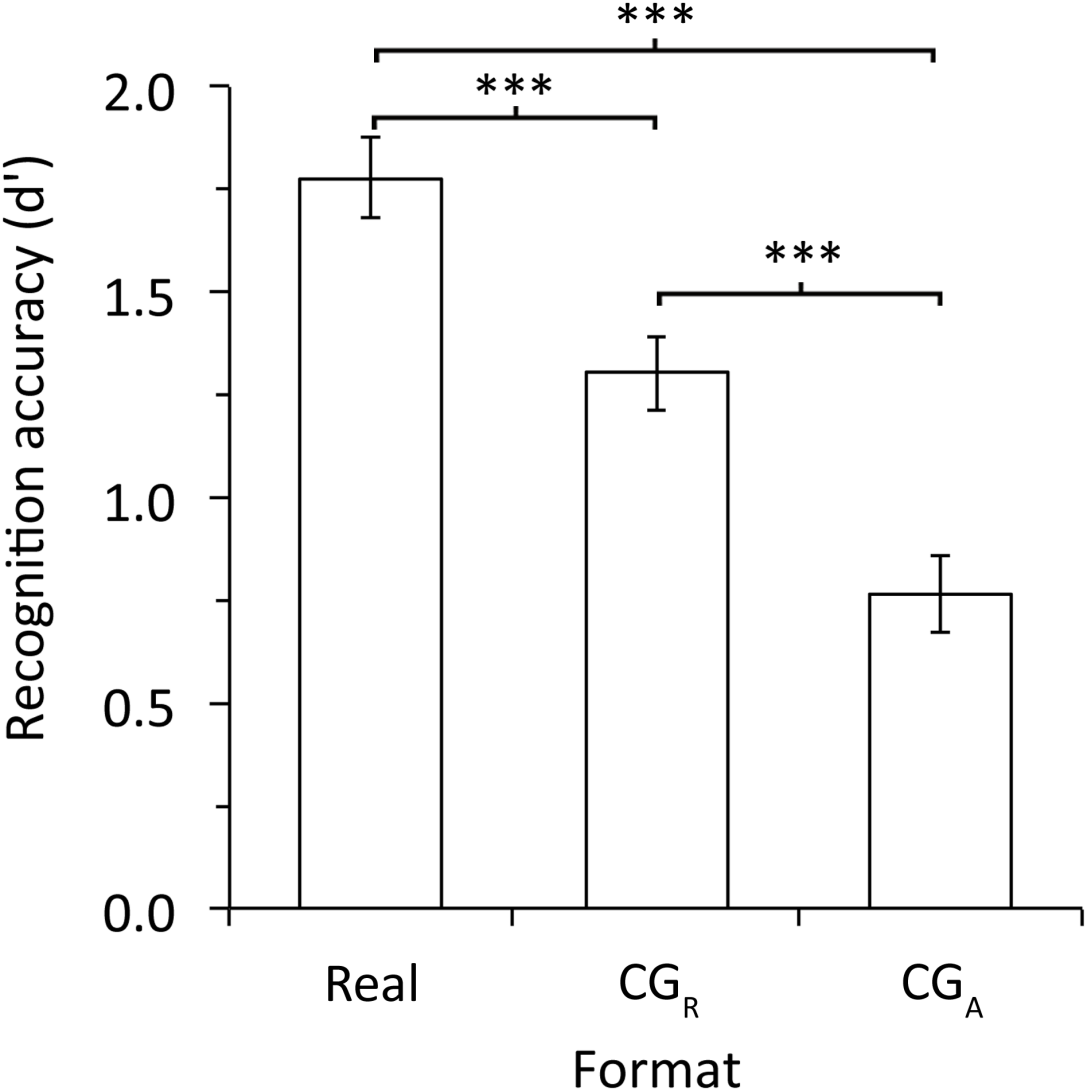
Experiment 1: recognition accuracy for own-race faces in the three face format conditions collapsed across race of participant. Error bars show ± 1 *SEM*. *** = *p* < .001.

Analysis of d' for own-race faces, the condition for which all participants should be experts, showed that CG faces were recognised less accurately than Real faces (see Fig 2). A mixed model ANOVA with format (Real, CG_R_, CG_A_) as the within participants factor and participant race (Caucasian, Asian) as the between participants factor revealed a significant main effect of format, *F*(2,138) = 38.01, *MSE* = 0.48, *p* <.001,η_p_^2^ = .36.Real faces were recognised significantly more accurately than CG_R_, *t*(70) = 5.08, *p* <.001,Cohen’s *d* = 0.60, and CG_A_ faces, *t*(70) = 7.65, *p* <.001,Cohen’s *d* = 0.91. CG_R_ faces were also recognised more accurately than CG_A_ faces, *t*(70) = 4.53, *p* <.001,Cohen’s *d* = 0.54. Reduced accuracy for the CG_R_ and CG_A_ faces is consistent with reduced expertise for CG faces. Finally, there was no main effect of participant race, *F*(1,69) = 0.01, *MSE* = 0.98, *p* = .944,η_p_^2^ = .00,and no interaction, *F*(2,138) = 0.63, *MSE* = 0.48, *p* = .534,η_p_^2^ = .01.

### Is the ORE reduced for CG faces?

**Fig 3:**
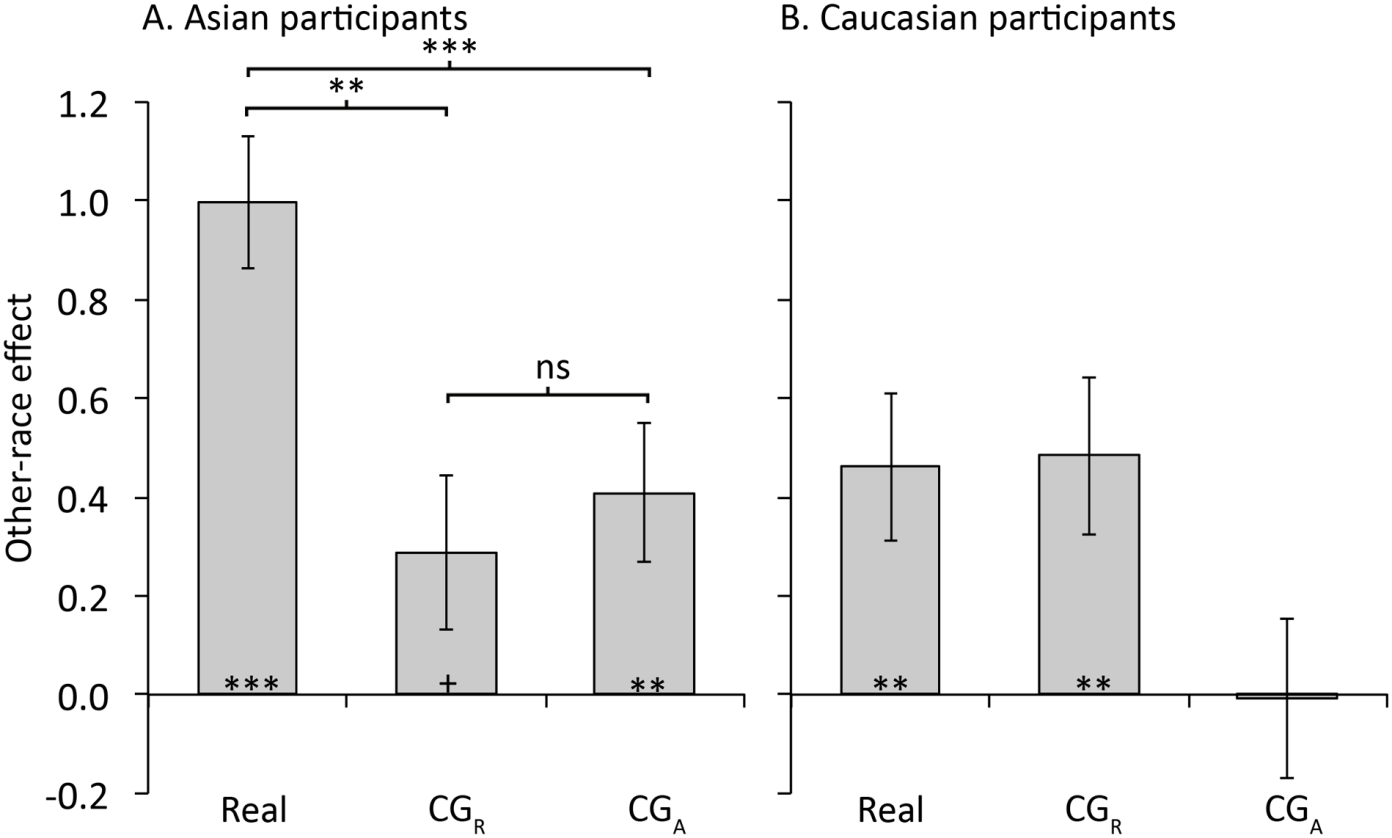
Experiment 1: Other-race effect (d' own-race minus d' other-race) as a function of format for A. Asian participants and B.Caucasian participants. Results of one sample significance tests of the ORE are shown at the base of the bars. Error bars show ± 1 *SEM*. *** = *p* < .001,** = *p* <.01,+= *p* = .07.

To compare the size of the ORE across format conditions an ORE score was calculated as d' own-race minus d' other-race. The ORE was reduced or eliminated for CG faces compared to Real faces (see Fig 3). As shown in Fig 3 this reduction was particularly evident in the CG_A_ condition, that is, for the type of CG face most widely used in face processing research. To confirm the observed differences in ORE a mixed model ANOVA was performed on the ORE scores with format (Real, CG_R_, CG_A_) as a within participants factor and participant race (Caucasian, Asian) as a between participants factor. There was a significant main effect of both format, *F*(2,138) = 6.33, *MSE* = 0.81, *p* = .002,η_p_^2^ = .08,and participant race, *F*(1,69) = 4.14, *MSE* = 0.82, *p* = .046,η_p_^2^ = .06. These effects were qualified by a significant format x participant race interaction, *F*(2,138) = 3.42, *MSE* = 0.81, *p* = .036,η_p_^2^ = .05.

To explore this interaction we conducted separate one-way ANOVAs for each race of participant. For Asian participants (Fig 3A) there was a significant main effect of format, *F*(2,68) = 7.07, *MSE* = 0.71, *p* = .002,η_p_^2^ = .17.Compared to Real faces the ORE was significantly smaller in both the CG formats: Real vs. CG_R_, *t*(34) = 3.46, *p* = .001,Cohen’s *d* = 0.58; Real vs. CG_A_, *t*(34) = 2.85, *p* = .007,Cohen’s *d* = 0.48. The size of the ORE was not significantly different between the two CG conditions, *t*(34) = 0.63, *p* = .533,Cohen’s *d* = 0.11. These results for Asian participants thus provide evidence that the ORE is reduced for CG compared to Real faces and suggest that CG faces do not fully recruit face expertise.

For Caucasian participants (Fig 3B) the main effect of format was only marginally significant, *F*(2,70) = 3.10, *MSE* = 0.89, *p* = .051,η_p_^2^ = .08. This result suggests that the size of the ORE was not smaller for CG compared to real faces for Caucasian participants.

If CG faces fail to recruit face expertise at all then we would expect the ORE to be absent for CG faces. To test this prediction one-sample t tests comparing the ORE to zero were performed for each format condition separately for each race of participant. Firstly we confirmed that the ORE was significant in the real condition for both Asian participants, *t*(34) = 7.39, *p* < .001,Cohen’s *d* = 1.25, and Caucasian participants, *t*(35) = 3.10, *p* = .004,Cohen’s *d* = 0.52. In the CG_R_ condition the ORE was significantly different from zero for the Caucasian participants, *t*(35) = 3.02, *p* = .005, Cohen’s *d* = 0.50, but was not for Asian participants, *t*(34) = 1.85, *p* = .073, Cohen’s *d* = 0.31. In the CG_A_ condition the reverse was true, the ORE was significantly different from zero for the Asian participants, *t*(34) = 2.88, *p* = .007, Cohen’s *d* = 0.49, but not for the Caucasian participants, *t*(35) = 0.05, *p* = .96, Cohen’s *d* = 0.01. These results provide evidence that the ORE was eliminated for CG faces in some cases but not in others.

Overall poor own-race face recognition and reductions in the ORE signal that CG faces may not fully recruit face expertise. This was particularly the case for CG_A_ faces, which are the more common class of CG faces used in face processing research.

## Experiment 2: Perceptual discrimination

In Experiment 2 we investigated whether the reduced own-race accuracy and reduced ORE for CG faces observed in Experiment 1 are restricted to recognition memory or also extend to perceptual discrimination. We used a simultaneous matching task in which participants had to match a target presented at the top of the screen to the same face identity in an array of 10 faces presented below the target [31]. On half the trials the target was not present in the array. A perceptual matching task including target absent trials was used to increase the difficulty of the task and because this task yields a clear ORE [22].

We compared matching performance for Real and CG_R_ faces. Given that these two formats contain the same identities the assignment of the same arrays to the Real or CG_R_ format could be counterbalanced across participants. The heterogeneity within the arrays has the potential to greatly affect accuracy on this task and could not be controlled at all in the CG_A_ format. If CG faces fail to fully recruit face expertise we expect to see reduced accuracy and a reduced ORE in the CG compared to the Real face condition.

## Method

### Participants

Caucasian participants were 30 students (17 male; Age: Mean = 18.8, SD = 3.2) at the University of Western Australia. Asian participants were 30 ethnically Chinese students or staff (4 male; Age: Mean = 20.7, SD = 3.3) at the University of Hong Kong. Participants received either course credit (Caucasian participants) or HK$60 (approximately US$8) for the 60 minute experiment.

### Stimuli

**Fig 4:**
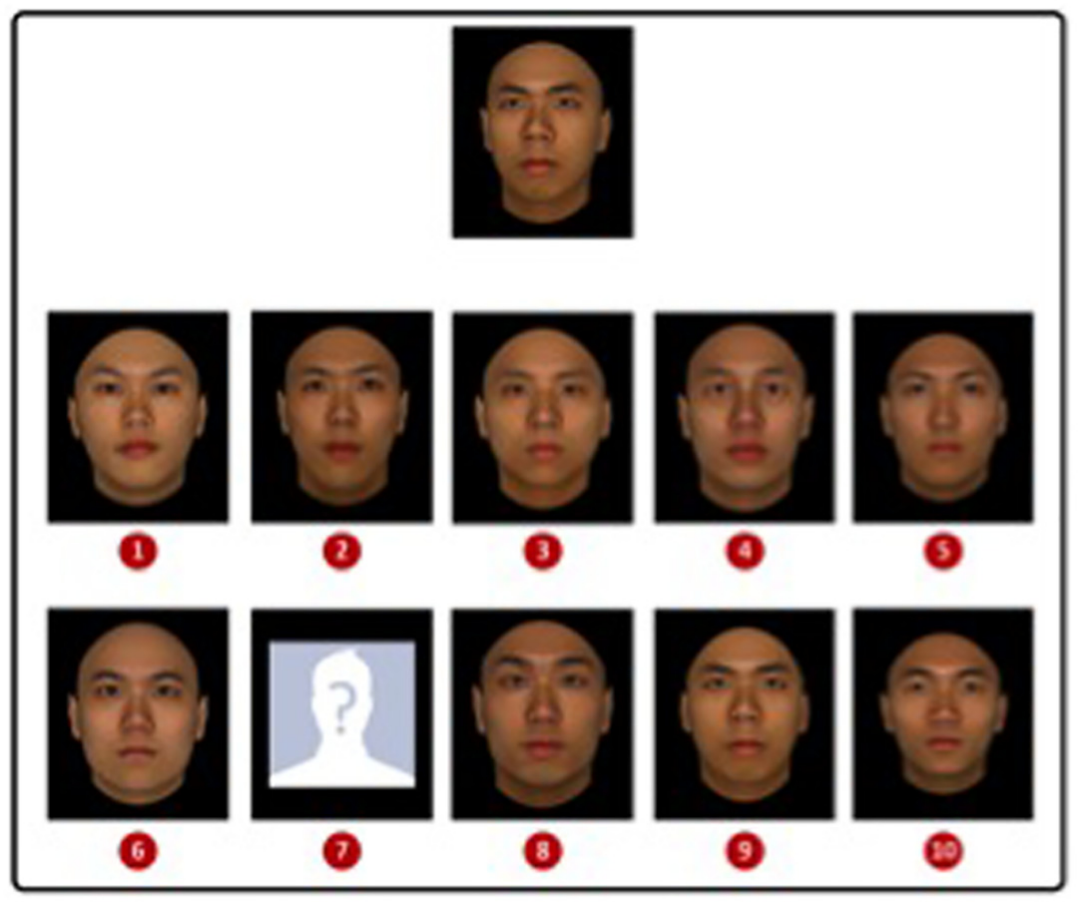
An example trial screen from Experiment 2 showing an Asian CG_R_ target present trial. Participants were required to identify the target depicted at the top of the screen in the array below. The correct response in this example is 9.

The stimuli were the 40 Real and 40 CG_R_ faces used in Experiment 1. In addition a “mystery man” stimulus [32], consisting of a silhouette of a head presented against a blue background with a question mark where the face should be (see Fig 4, position 7), was created for use as an item in the arrays for the “target absent” response. Each face was pasted on a black square (see Fig 4) measuring 5.9 cm horizontal by 6.9 cm vertical. Face stimuli were an average of 5.2° horizontal (ear to ear) by 6.5° vertical (top of visible forehead to bottom of visible neck) at the viewing distance of approximately 50 cm.

Each trial display consisted of a target face presented at the top of the screen with an array of faces presented below it (see Fig 4 for an example trial display). Arrays consisted of 9 faces of the same race as the target plus the “mystery man”. Targets were 5° left views. Faces in the array were all front view.

### Procedure

There were two format conditions: Real, CG_R_; and face formats were intermixed in a different random order for each participant. Note—there were two additional conditions in which the format of the target was different to that of the array (i.e., Real target with CG_R_ array and CG_R_ target with Real array). Results from these conditions are not theoretically interesting and are therefore not reported. The 40 faces of each race were divided into four sets of 10 faces. For each participant one set was assigned to the Real condition and another to the CG_R_ condition. The particular set assigned to each condition was counterbalanced across participant according to a Latin square.

The participant’s task was to identify the target in the array. Within each format condition half of the trials were “target absent”, that is the target did not appear in the array. The position in the array of the target/mystery man was randomly assigned on each trial.

There were 20 Real trials and 20 CG trials. Each face in the set appeared as the target in only one trial. Whether a particular face appeared as the target in a target absent or a target present trial was counterbalanced across participants. Each face appeared as a distractor (non-target) in the array in 7–10 trials.

The task was run using PsyScope X [33] on the same computers as Experiment 1. Each trial was initiated by the participant pressing the spacebar. A target and array then appeared simultaneously and remained visible until response. Participants entered the number corresponding to the selected face on a keyboard (the zero key was relabelled 10). Following the response to each array a prompt appeared asking participants to rate “How sure are you?” from 1 (“completely guessing”) to 5 (“completely sure”). Participants entered confidence ratings using the keyboard.

Participants were told that there was no time limit and that they were to respond as accurately as possible. They were also informed that on about half the trials the target would be absent, in which case they were to select the “mystery man”. To encourage participants to try to perform as accurately as possible we displayed the top 10 scores on the task in the testing room. Participants were told they could find out their own score at the completion of the task and add it to the leader-board if they qualified. Self-timed breaks were provided every 20 trials.

To familiarise participants with the task procedure they first completed a practice phase using characters from the *The Simpsons* as stimuli. There were 8 practice trials (4 target present, 4 target absent). No feedback was provided.

Following the discrimination task participants completed the racial background and contact questionnaire and the CG-face experience questionnaire described in Experiment 1. Unfortunately, due to experimenter error the racial background and contact questionnaire was not collected from one Caucasian participant and the CG-face experience questionnaire was not collected from seven Caucasian participants.

## Results and Discussion

### Contact

Self-reported contact with own-race, other-race and CG faces was calculated as in Experiment 1 (see Table 3). Again, as expected, both groups of participants reported significantly greater contact with own-race than other-race faces: Caucasian participants, *t*(28) = 7.62, *p* < .001, Cohen’s *d* = 1.41; Asian participants, *t*(29) = 15.43, *p* < .001, Cohen’s *d* = 2.82. There was no difference between the groups in reported experience with CG faces, *t*(51) = 0.61, *p* = .544, Cohen’s *d* = 0.17.

**Table 3:** Experiment 2: Mean (SD) self-reported contact.

	Own-race	Other-race	CG
Caucasian participants	5.2 (0.4)	4.0 (0.8)	2.6 (0.8)
Asian participants	5.3 (0.5)	2.4 (0.8)	2.7 (0.7)

### Discrimination accuracy

Accuracy for target present and target absent trials was calculated for each condition (see Table 4). Results from the confidence measure showed a similar pattern to the results for accuracy (i.e., in conditions where participants were more accurate they were also generally more confident) and accuracy and confidence were correlated in most conditions (Tables B and C in S1 File for details).

**Table 4:** Experiment 2: Mean (SD) face recognition accuracy (% correct) for target present (TP) and target absent (TA) trials as a function of participant race, race of face and face format.

	Real		CG_R_	
Target status	TP	TA	TP	TA
Caucasian Participants				
own-race faces	94.0 (10.7)	79.3 (23.2)	90.0 (17.2)	53.3 (33.8)
other-race faces	90.0 (15.5)	66.7 (30.3)	86.0 (19.1)	45.3 (31.0)
other-race effect	4.0 (15.2)	12.7 (27.53)	4.0 (18.5)	8.0 (30.9)
Asian Participants				
own-race faces	94.7 (11.7)	86.7 (19.2)	92.0 (11.3)	60.0 (33.6)
other-race faces	96.0 (8.1)	83.3 (22.3)	91.3 (14.6)	58.0 (31.7)
other-race effect	-1.3 (14.8)	3.3 (17.5)	0.7 (17.8)	2.0 (32.9)

### Are own-race CG faces matched less accurately than real own-race photographs?

**Fig 5:**
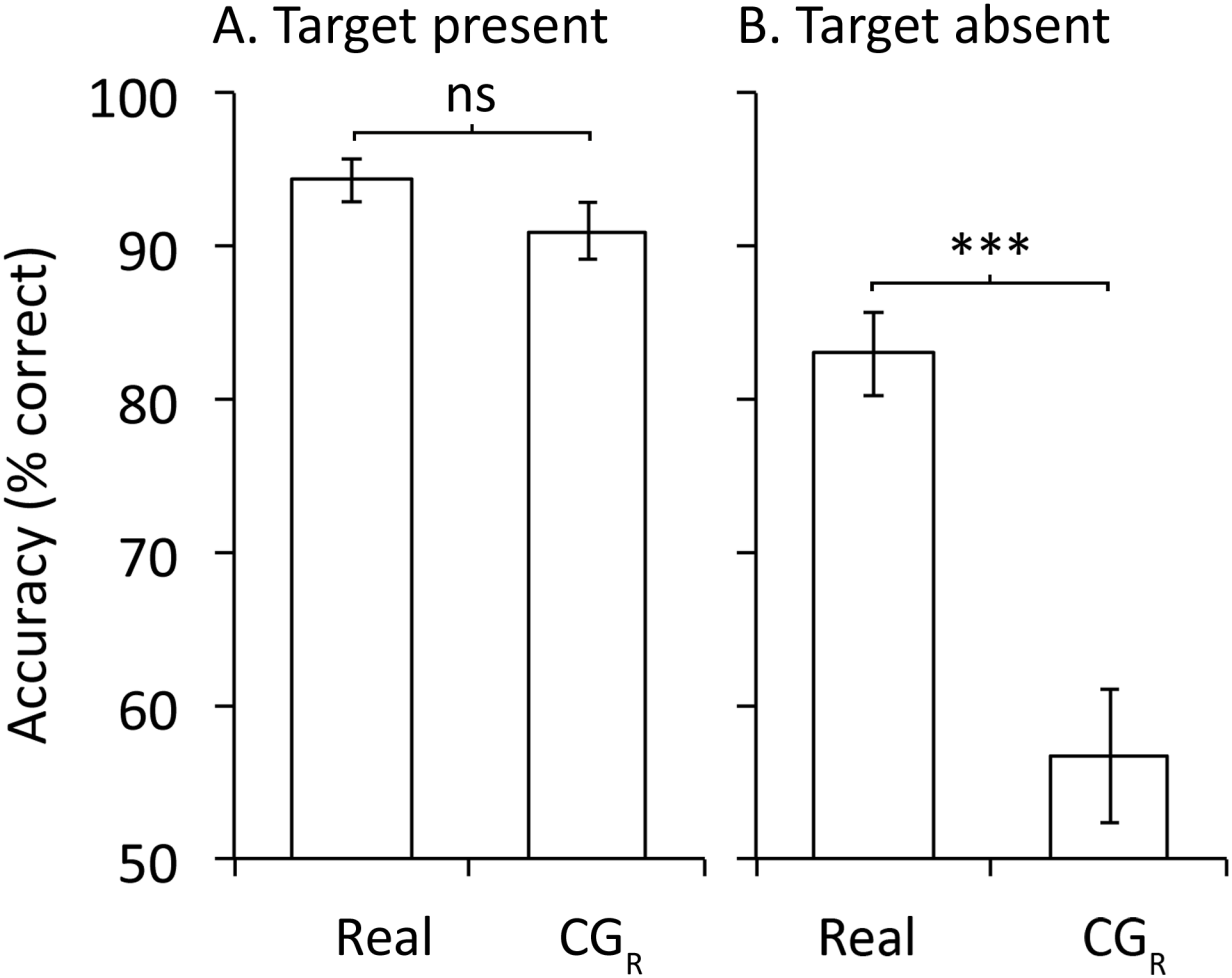
Experiment 2: discrimination accuracy for own-race faces in the two format conditions collapsed across race of participant for A. target present trials and B. target absent trials. Error bars show ± 1 *SEM*. *** = *p* < .001.

CG_R_ faces were matched less accurately than Real faces and this effect was much larger for target absent than target present trials (see Fig 5). A format (Real, CG_R_) x target presence (Present, Absent) x participant race (Caucasian, Asian) ANOVA revealed a significant main effect of format, *F*(1,58) = 35.95, *MSE* = 367.18, *p* < .001, η_p_^2^ = .38, reflecting greater accuracy for Real than CG_R_ faces. However there was also a significant main effect of target presence, *F*(1,58) = 54.75, *MSE* = 571.32, *p* < .001, η_p_^2^ = .49, and format x target presence interaction, *F*(1,58) = 29.98, *MSE* = 264.66, *p* < .001, η_p_^2^ = .33. No other effects or interactions were significant, all *F*s < 1.5, all *p*s > .2.

Following up this interaction, in the target present condition (see Fig 5) there was no difference in accuracy between the Real and the CG_R_ faces, *t*(59) = 1.52, *p* = .13, Cohen’s *d* = 0.26. As can been seen in Fig 5 accuracy in the target present condition was close to ceiling for both races of face—if the target was present in the array then participants could accurately identify him. This ceiling effect may have masked an effect of format on target present trials. However, on target absent trials (Fig 5B), where performance was well below ceiling, format had a large effect. Participants were significantly more accurate at reporting that the target was not in the array for Real than CG_R_ faces, *t*(59) = 6.60, *p* < .001, Cohen’s *d* = 0.94. This reduced accuracy for CG compared to Real faces in perceptual discrimination, in the target absent trials, mirrors the result for recognition memory in Experiment 1. Once again this result is consistent with reduced expertise for CG faces.

### Is the face matching ORE reduced for CG faces?

To address this question an ORE score (% correct own-race minus % correct other-race) was calculated for target present and target absent trials in each condition (Table 4). The ORE was not reduced for CG_R_ compared to Real faces. A format (Real, CG_R_) x target presence (Present, Absent) x participant race (Caucasian, Asian) ANOVA revealed only a significant main effect of participant race, *F*(1,58) = 4.08, *MSE* = 528.85, *p* = .048, η_p_^2^ = .07, with Caucasian participants demonstrating a larger ORE (*M* = 7.2, *SD* = 11.5) than Asian participants (*M* = 1.2, *SD* = 11.5). No other effects or interactions were significant, all *F*s < 2.06, all *p*s > .15. The lack of a main effect of format suggests that the ORE was not different between Real and CG_R_ conditions.

Overall, reduced matching accuracy for own-race CG faces suggests a failure to fully recruit face expertise but this was not reflected in the ORE results.

## General Discussion

The results of this study provide important, new evidence that CG faces do not allow participants to demonstrate the full extent of their face expertise. This conclusion is based on the finding that two key markers of face expertise were diminished for CG compared to Real faces. In Experiment 1 recognition memory accuracy was significantly poorer for CG than Real own-race faces. The ORE was also significantly reduced or eliminated for CG compared to Real faces in three out of four conditions. In Experiment 2 perceptual matching accuracy on target absent trials was significantly worse for CG compared to Real own-race faces. Here, however, no differences between formats were found for the ORE. In combination these results suggest caution should be applied when using CG faces to examine expert processing of face identity.

Our finding that CG_R_ faces generated from real photographs were more difficult to remember than real faces supports two previous findings [4, 25] for such faces. Further, in a comparison not previously tested, we found that randomly generated CG_A_ faces were even more poorly remembered than the CG_R_ faces. Our finding that perceptual discrimination was also poorer for CG_R_ compared to real faces on target absent trials supports a similar recent finding from Balas and Pacella [25] using a delayed match to sample task. Together these results argue that CG faces do not allow participants to demonstrate the full extent of their face recognition abilities.

The results for the ORE were less straightforward. For Asian participants the ORE on recognition memory was significantly reduced for both CG_R_ and CG_A_ faces suggesting a failure to fully tap face expertise. However for the Caucasian participants the ORE on recognition memory was not significantly smaller for CG compared to real faces (see also [4]) although note that the ORE eliminated for CG_A_ faces. Similarly no effects of format were observed on the perceptual matching ORE. These results suggest caution when interpreting results using CG faces as the effects may be smaller than would have been observed for real faces. These results also highlight the importance of testing both races of participants. Had we, for example, only tested Caucasian participants in both experiments our conclusions would have been different.

Why might these CG faces fail to fully reveal face expertise? We propose three possible explanations. First, it could be that CG faces objectively contain less discriminating information, and are therefore more similar to each other, than real faces. The CG faces used here, especially the wholly artificial faces (CG_A_), clearly lack fine-grained surface texture and small scale variations in colour information usually seen in photographs of faces. Surface information is important for own-race face recognition (e.g., [14, 34]) and a lack of discriminating surface information can reduce the ORE [35]. These results suggest that the use of surface information is an important aspect of face expertise and that the loss of this information has the greatest effect on faces for which we are most expert: own-race faces. A reduction in surface information may therefore explain both the reduced own-race CG face accuracy and the reduced ORE. It is less clear from simple inspection whether or not structural shape information is also impoverished in CG faces, but any loss of distinguishing shape information could also contribute to a reduction in own-race accuracy and the ORE [35]. Thus, CG faces contain less surface information and possibly less shape information than real faces, both of which could contribute to the failure to fully recruit face expertise indicated by our results.

Second, it is possible that real and CG faces contain comparable amounts of discriminating information but that our face processing mechanisms are simply less well tuned to the variation present in CG faces, just as they are less well tuned to the variation in other-race faces (see [36] for review). On average, our participants reported relatively little exposure to CG faces and the exposure they did have was unlikely to have been with faces of precisely the type used in this experiment. In this way the CG faces in our experiment may have been analogous to less-experienced other-race faces. On this view, sufficient information would be available for accurate recognition of CG faces but our face processing mechanisms are not using it efficiently because they are not optimally tuned to it. If this is correct, then with more CG face experience participants could potentially recognise CG faces just as well as own-race real faces.

Third, given that people are very sensitive to deviations from animacy [10] and that they can have aversive reactions to human-like CG faces (the “uncanny valley” effect e.g., [37, 38]), it is conceivable that the CG faces were classed as out-group faces (i.e., “not human” or “inanimate”), which are recognised less well than in-group faces (e.g., rival university vs. own university, [39]). If all CG faces were considered out-group faces, then reduced accuracy and a lack of differentiation between own- and other-race faces would be expected.

Regardless of the underlying cause, we suggest that, as currently generated, CG faces seem poorly suited for investigating expert processing of face identity. Just as researchers would not be advised to use other-race face stimuli when investigating expert face recognition, the results of the present study raise concerns about the use of these types of CG faces. Note that at this stage our conclusions specifically apply to the type of CG faces used in this experiment (i.e., FaceGen stimuli both wholly artificial and generated from photographs) and may not generalise to other sources of CG faces. We also note that having tested in two different countries our results generalise beyond a single population but the extent to which our findings generalise beyond the typical, university educated adult population tested here is unknown.

The type of CG faces used in the present study are being commonly used in face processing research. Our conclusions therefore have implications for studies that have used these CG faces as stimuli. For example, studies that have used CG faces to investigate populations with face recognition difficulties, such as prosopagnosia, may have under-estimated the extent of any deficits, because typical participants may be underperforming due to the use of CG faces [40–42]. Similarly the effectiveness of treatments may be over-estimated if performance in the prosopagnosic group is improved in relation to an underperforming control group [40]. In another example, studies that have failed to show effects may not have given the face system the full opportunity to demonstrate them, that is, effects may have been present if photographs had been used. For example, manipulations that have produced a reduction or elimination of the ORE [2, 43] may not have done so if real faces, which can produce a stronger ORE, had been used.

It is important to stress that the current findings do not mean that there is no place for CG faces in research. Rather, we propose that there needs to be greater awareness and acknowledgement of the potential limitations of such stimuli. Face expertise has a number of facets, only one of which was tested here: identity. It remains an open question whether CG faces are suitable for investigating other aspects of face processing, such as participants’ ability to read emotional expression, gaze direction, and personality characteristics like trustworthiness or dominance. Future studies could also assess whether CG faces are appropriate for testing other markers of face identity expertise such as holistic processing. It is possible that effects associated with holistic processing, such as the composite and part-whole effects, would also be attenuated for CG compared to real faces. Additionally, CG faces can be useful when researchers are not specifically interested in expert processing. For example, CG faces have been used to explore the “exposure duration effect” where stimuli presented for a longer duration are rated as more attractive [44]. In this study the authors were not interested in face expertise per se, but FaceGen provided a convenient method of generating highly standardised stimuli for exploring the effect of interest.

Finally, we note that our conclusions are based entirely on the state of one piece of current software. Further technological advances may produce software in the future that can generate faces that do fully demonstrate expert face processing. Such software would be an invaluable resource for face researchers. However reaching this point may require the production of CG faces that are indistinguishable from photographs.

## Supporting Information

S1 File Table A, *Experiment 1*: *Mean (SD) proportion hits and false alarms (FA) as a function of participant race*, *race of face and face format*. Table B, *Experiment 2*: *Mean (SD) confidence for target present (TP) and target absent (TA) trials as a function of participant race*, *race of face and target-array format condition*. *Confidence was rated on a 5 point scale (1 = completely guessing*, *5 = completely sure*. Table C, *Experiment 2*: *Correlation (r) between mean accuracy and mean confidence*.
(DOCX)
