# Supplementary material for: How Well Do Computer-Generated Faces Tap Face Expertise?
Source: PLoS One. 2015 Nov 4;10(11):e0141353. doi: 10.1371/journal.pone.0141353 (PMC4633121; doi:10.1371/journal.pone.0141353)
Supplement: S1 File — Table A, Experiment 1: Mean (SD) proportion hits and false alarms (FA) as a function of participant race, race of face and face format. Table B, Experiment 2: Mean (SD) confidence for target present (TP) and target absent (TA) trials as a function of participant race, race of face and target-array format condition. Confidence was rated on a 5 point scale (1 = completely guessing, 5 = completely sure. Table C, Experiment 2: Correlation (r) between mean accuracy and mean confidence. (DOCX) [file pone.0141353.s001.docx]

**Supplementary Materials**

**Experiment 1: Hits and false alarms**

Table A

*Experiment 1: Mean (SD) proportion hits and false alarms (FA) as a function of participant race, race of face and face format.*

| Format | Real | | CG_R_ | | CG_A_ | |
| --- | --- | --- | --- | --- | --- | --- |
|  | Hits | FA | Hits | FA | Hits | FA |
| Caucasian Participants |  |  |  |  |  |  |
| own-race faces | 0.82 (0.13) | 0.27 (0.16) | 0.76 (0.16) | 0.32 (0.18) | 0.69 (0.17) | 0.44 (0.20) |
| other-race faces | 0.75 (0.16) | 0.33 (0.16) | 0.69 (0.17) | 0.40  (0.17) | 0.68 (0.16) | 0.43 (0.14) |
| Asian Participants |  |  |  |  |  |  |
| own-race faces | 0.78 (0.15) | 0.21 (0.15) | 0.71 (0.14) | 0.31 (0.17) | 0.67 (0.19) | 0.41 (0.19) |
| other-race faces | 0.67 (0.16) | 0.39 (0.19) | 0.73 (0.13) | 0.40 (0.20) | 0.66 (0.15) | 0.51 (0.17) |

**Experiment 2: Confidence results**

Mean confidence for target present and target absent trials was calculated for each condition (see Table A). In general, in the conditions in which participants were less accurate they were also less confident in their responses.

Table B

*Experiment 2: Mean (SD) confidence for target present (TP) and target absent (TA) trials as a function of participant race, race of face and target-array format condition. Confidence was rated on a 5 point scale (1 = completely guessing, 5 =*

*completely sure.*

|  | Real | | CG_R_ | |
| --- | --- | --- | --- | --- |
| Target status | TP | TA | TP | TA |
| Caucasian Participants |  |  |  |  |
| own-race faces | 4.6 (0.5) | 3.6 (0.7) | 4.5 (0.6) | 3.4 (0.8) |
| other-race faces | 4.5 (0.6) | 3.6 (0.7) | 4.4 (0.5) | 3.6 (0.8) |
| Asian Participants |  |  |  |  |
| own-race faces | 4.8 (0.4) | 3.8 (0.7) | 4.6 (0.5) | 3.5 (0.6) |
| other-race faces | 4.6 (0.5) | 3.7 (0.7) | 4.5 (0.5) | 3.4 (0.6) |

**Own-race confidence**

The increase in difficulty for CG_R_ compared to Real faces found for accuracy was also reflected in the confidence measure. A format (Real, CG_R_) x target presence (present, absent) x participant race (Caucasian, Asian) ANOVA produced a significant main effect of format, *F*(1,58) = 14.24, *MSE* = 0.12, *p* < .001, η_p_^2^ = .20, reflecting greater confidence in the Real (*M* = 4.2, *SD* = 0.4) than the CG_R_ condition (*M* = 4.0, *SD* = 0.4). Participants were also more confident on target present (*M* = 4.6, *SD* = 0.3) than target absent trials (*M* = 3.6, *SD* = 0.4), *F*(1,58) = 330.85, *MSE* = 0.19, *p* < .001, η_p_^2^ = .85. There were no other significant effects or interactions, all *F*s < 1.7, *p*s > .2.

**ORE**

A confidence ORE score (confidence own-race minus confidence other-race) was calculated for target present and target absent trials in each condition. There was no evidence of a reduction in the confidence ORE for CG compared to Real faces. A format (Real, CG_R_) x target presence (present, absent) x participant race (Caucasian, Asian) ANOVA revealed only a main effect of participant race, *F*(1,58) = 4.78, *MSE* = .26, *p* = .033, η_p_^2^ =.08, with Asian participants demonstrating a larger ORE (*M* = 0.14, *SD* = 0.25) than Caucasian participants (*M* = 0.00, *SD* = 0.25). There were no other significant effects or interactions, all *F*s < 1.9, *p*s > .17.

**Correlations between Accuracy and Confidence**

Correlations between mean accuracy and mean confidence were calculated for each condition collapsed across race of participant. As seen in Table C accuracy was significantly correlated with confidence in most conditions. Most notably the correlations between accuracy and confidence were not significant for CG faces on Target Absent trials. This result again suggests that the CG faces were not treated in the same way as real photographs. However these correlations were significantly different from those for Real faces only in the own-race condition, *z*(60) = 2.43, *p* = 0.02, but not in the other-race condition, *z*(60) = 1.00, *p* = 0.32, (z calculated using formulas from Raghunathan, Rosenthal, & Rubin, 1996).

*Table C*

*Experiment 2: Correlation (r) between mean accuracy and mean confidence*

|  | Real | | CG_R_ | |
| --- | --- | --- | --- | --- |
| Target status | TP | TA | TP | TA |
| All participants |  |  |  |  |
| own-race faces | .176 | .373** | .635** | -.023 |
| other-race faces | .356** | .304** | .450** | .152 |

**References**

Raghunathan, T. E., Rosenthal, R., & Rubin, D. B., (1996). Comparing correlated but nonoverlapping correlations. *Psychological Methods, 1*, 178-183.
